# Tritium-Labeled Compounds IV. d-Glucose-*6*-*t*, d-Xylose-*5*-*t*, and d-Mannitol-*1*-*t*

**DOI:** 10.6028/jres.064A.037

**Published:** 1960-08-01

**Authors:** Horace S. Isbell, Harriet L. Frush, Joseph D. Moyer

## Abstract

Methods are presented for the preparation of d-glucos*e-6-t*, d-xylose-*5-t*, and d-mannitol-*1*-*t* by the reduction of suitable compounds with lithium borohydride-*t* in anhydrous tetrahydrofuran, followed by hydrolysis of the products. The starting materials for the reductions are, respectively, 1,2-*O*-isopropylidene-d-glucurono-6,3-lactone, 5-aldo-1,2-*O*-isopropylidene-d-*xylo*-pentofuranose, and 2,3:5,6-di-*O*-isopropylidene-d-mannofuranose. The apparatus and procedure for carrying out the reductions in a closed system are described.

## 1. Introduction and Discussion

This report is one of a series on the production and use of tritium-labeled carbohydrates.^1^ Previous papers from this laboratory have described convenient apparatus for handling tritium in a closed system [2],^2^ and methods for analyzing nonvolatile tritium compounds [3, 4]. A method for preparing lithium borohydride-*t* [2], a versatile reductant for introducing tritium into organic compounds, was also given. The usefulness of this compound, in aqueous pyridine, for preparing position-labeled carbohydrates has already been demonstrated [5]; this paper describes its use in the reduction of certain carbohydrate derivatives in an anhydrous solvent.

The equations illustrate the preparation of d-glucose-*6*-*t*, d-xylose-*5-t*, and d-mannitol-*1*-*t* by reduction of suitable compounds with lithium borohydride-*t* in anhydrous tetrahydrofuran. Previously, Sowden had reduced 1,2-*O*-isopropylidene-d-glucurono-6,3-lactone (I) in the preparation of d-glucose-*6-C*^14^ [6]. The reduction, originally carried out with sodium borohydride in water, was improved by Roseman, who used lithium aluminum hydride in anhydrous ether [7]. Crystalline 5-aldo-1,2-*O*-isopropylidene-d-*xylo*-pentofuranose (II) [8, 9] was prepared as a starting material for the synthesis of d-glucose-*6*-*C*^14^ [8]. Earlier, Sowden had reduced this substance (II), prepared from d-glucose-*1*-*C*^14^, with hydrogen and Raney nickel and obtained d-xylose-*1*-*C*^14^ [10]. Reduction of 5-aldo-1,2-*O*-isopropylidene-d-*xylo*-pentofuranose with lithium borohydride-*t* in tetrahydrofuran, and subsequent hydrolysis, yields d-xylose-*5*-*t*. The reduction of 2,3:5,6-di-*O*-isopropylidene-d-mannofuranose (III) [11] has been used in this laboratory for several years as a means for analyzing lithium borohydride-*t*. The reduction takes place nearly quantitatively, and the specific activity of the d-mannitol-*1*-*t*, readily obtained on hydrolysis, can be used as a measure of the specific activity of the lithium borohydride-*t*.

**Figure:**
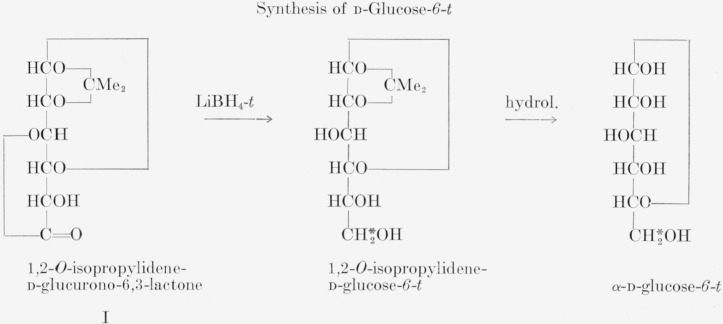

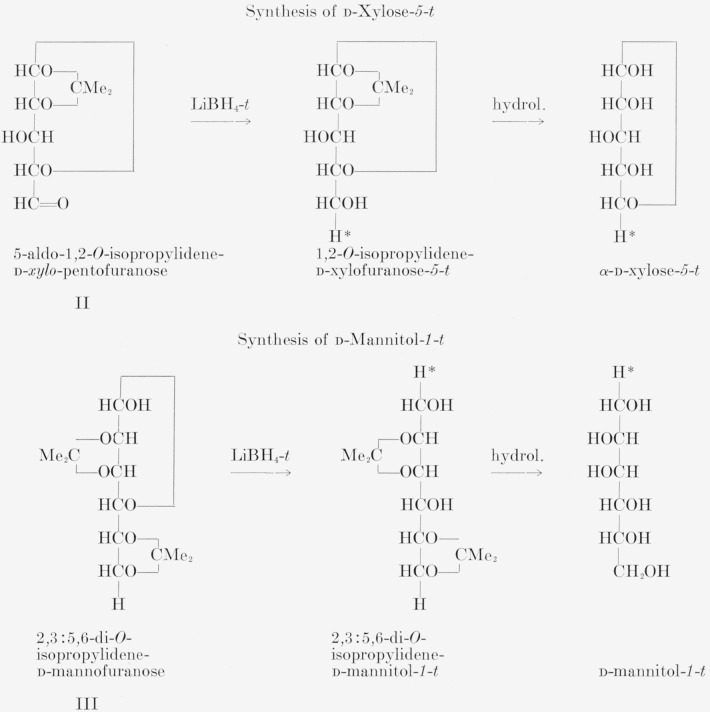


## 2. Experimental Details

### 2.1. Apparatus and Materials

**Figure 1:**
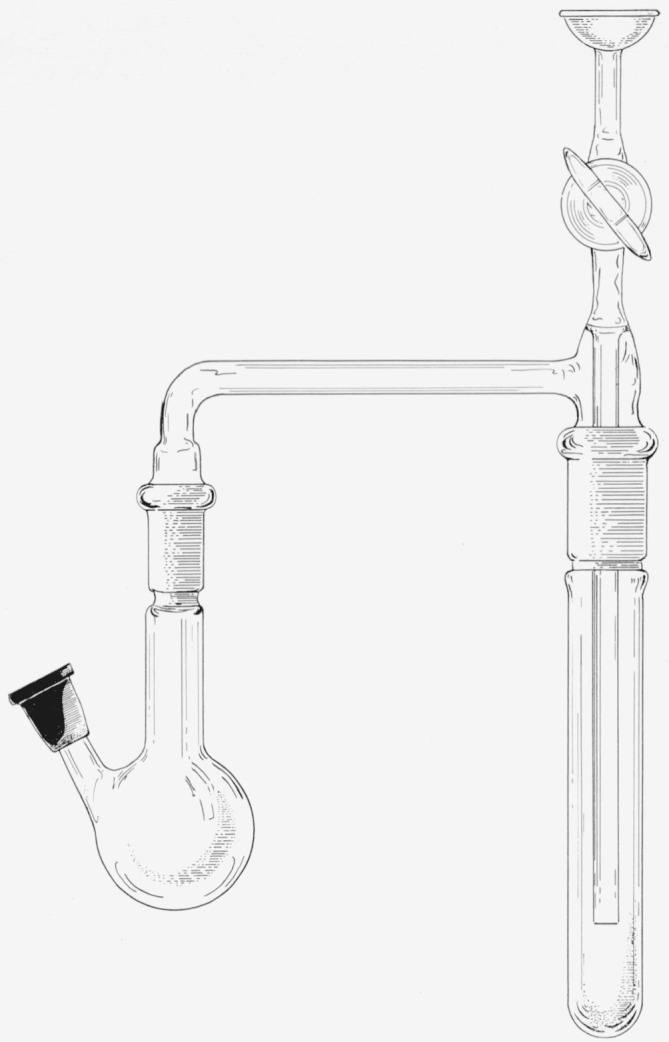
Apparatus for conducting reductions with lithium borohydride-*t*. *A*, A 50-ml, round-bottomed reaction flask having a rubber-capped side-arm for introducing reagents. *B*, Trap for use in cooling-bath.

The general-purpose manifold and apparatus for working with tritium in a closed system are described in [2]. The reaction flask, used in conjunction with the manifold in the preparations described here, is shown in figure 1. The following starting materials for the reductions were prepared by the methods given in the references cited: 2,3-*O*-isopropylidene-d-glucurono-6,3-lactone [6], 5-aldo-1,2-*O*-isopropylidene-d-*xylo*-pentofuranose [8], 2,3:5,6-di-*O*-isopropylidene-d-mannofuranose [11], and lithium borohydride-*t* [2]. Anhydrous tetrahydrofuran and solutions of lithium borohydride-*t* therein were prepared by the methods previously described [2].

### 2.2. Analysis of Tritium Compounds

All analyses of tritium-containing materials were made with a 2*π*, windowless, gas-flow, proportional counter. Low-activity materials were assayed by the method of Isbell and coworkers [3], in films of sodium *O*-(carboxymethyl) cellulose (CMC) with the proportional counter operated at 2,000 v. The films were made on 2-in., stainless-steel planchets and counted to a statistical error of less than 1 percent (more than 10,000 counts). The specific activity of the material, *s*, in *μ*c/mg, was obtained from the relationship:

s=(mak)/m′,

where *a* is the observed counts per second, corrected for background, *m* is the total solids in the CMC film, *m*′ is the weight of the radioactive sample, and *k* is an empirical factor (4.45×10^−5^) independently determined under the conditions employed. High-activity materials were assayed in formamide solutions,^3^ and the proportional counter was operated at 1,750 v. Under the conditions used, 1 count per second corresponds to 0.128 *μ*c of tritium per milliliter of the formamide solution counted.

### 2.3. *α*-d-Glucose-*6*-*t*

Two millimoles of 1,2-*O*-isopropylidene-d-glucurono-6,3-lactone (432 mg) and a magnetic stirring bar were placed in flask A of figure 1. The flask and trap were connected to the general-purpose manifold of [2], and the system was made vacuum-tight and evacuated. The connection to the manifold was then closed, and the reaction flask was cooled in a shallow ice-bath set on a magnetic stirrer. The stirrer was started, and 5 ml of anhydrous tetrahydrofuran was injected into the flask by means of a hypodermic needle; this was followed by a solution of 2.0 millimoles of lithium borohydride-*t* (having a total of 80 mc of radioactivity) in approximately 4 ml of anhydrous tetrahydrofuran. After the solution had remained for 3 hr at 0°, 5 ml of water was injected, and the mixture was allowed to stand at room temperature overnight. The reaction mixture was then frozen in liquid nitrogen, and the hydrogen-*t* that was present was removed through the manifold. The mixture was warmed to room temperature, and 5 ml of water containing 1 millimole of nonradioactive sodium borohydride was added (in order to reduce any aldehyde groups that had escaped reduction with the lithium borohydride-*t*). After 30 min, the solution in flask A was frozen, and freeze-dried by cooling B in a dry-ice bath and evacuating the system through the manifold. Water was added to the residue by means of a hypodermic needle, and the solution was again freeze-dried. This process was repeated once more.

Finally, flask A was removed from the system, and the residue was dissolved in 20 ml of 1-percent hydrochloric acid. The solution was heated for 30 min in a boiling-water bath in order to hydrolyze the isopropylidene group, and was then passed through 20 ml of mixed anion-and cation-exchange resins.^4^ The effluent was concentrated in a rotary still, and all boric acid was removed as methyl borate by repeated addition and evaporation of methanol. The resulting product was dissolved in water, and the solution was passed through a column containing 5 ml of mixed anion-and cation-exchange resins. The conductivity of the solution, measured with a purity meter,^5^ was found to be low, in accordance with the absence of ionic impurities. A radioassay of an aliquot of the solution in formamide showed the presence of 33.5 mc of radioactivity in the product. The solution was evaporated almost to dryness in a rotary vacuum still. The residue was dissolved in 1 ml of methanol, and 2-propanol was added almost to the point of incipient turbidity. Crystallization of *α*-d-glucose-*6*-*t* was induced by seeding with the nonradioactive sugar. The crystals were separated, recrystallized, and assayed in a CMC film by means of the proportional counter. The product weighed 289 mg and had an activity of 29.5 mc. By cocrystallization of the mother liquor with nonradioactive d-glucose, about 2 mc of *α*-d-glucose-*6-t* of lower activity was recovered. Thus, the yield of d-glucose-*6*-*t*, based on the 1,2-*O*-isopropylidene-d-glucurono-6,3-lactone, was 85.7 percent; the radiochemical yield (31.5 mc) was 39.4 percent, based on the lithium borohydride-*t*.

### 2.4. *α*- -Xylose-*5*-*t*

Four millimoles of crystalline 5-aldo-1,2-*O*-isopropylidene-d-*xylo*-pentofuranose were reduced with 2 millimoles of lithium borohydride-*t* (containing 63.5 mc of radioactivity) by the technique described for the preparation of d-glucose-*6*-*t.* A crystalline substance, presumably 1,2-*O*-isopropylidene-d-*xylo*-pentofuranose-*5*-*t*, separated after the reduction. This intermediate compound was not purified, but was converted to d-xylose-*5*-t by heating it with 1-percent hydrochloric acid in a boiling-water bath for 1 hr. The solution was de-ionized in the manner previously described, and an aliquot was counted in formamide; the solution contained 26.1 mc of radioactivity. The labeled sugar was crystallized by concentrating the solution to a sirup and diluting it with ethanol and 2-propanol. After recrystallization, the *α*-d-xylose-*5-t* weighed 450 mg and had an activity of 53 *μ*c/mg. The yield, based on the weight of the 5-aldo-1,2-*O*-isopropylidene-d-*xylo*-pentofuranose, was 75 percent; the radiochemical yield was 38 percent.

### 2.5. d-Mannitol-*1-t*

One millimole of 2,3:5,6-di-*O*-isopropylidenc-d-mannofuranose (260 mg) was reduced with 0.5 millimole of lithium borohydride-*t* containing 16 mc of tritium, by the techniques described in section 2.3. The crystals that separated were recrystallized from water, with the addition of methanol. The purified d-mannitol-*1-t* weighed 175 mg and had an activity of 43 *μ*c/mg. The chemical yield was 95 percent, and the radiochemical yield, 31.3 percent.

## Footnotes

1 Project sponsored by the Division of Research of the Atomic Energy Commission. See [1] for a general description of the work.

2 Figures in brackets indicate the literature references at the end of this paper.

3 The method for radioassay in formamide solutions was developed earlier in this laboratory for the assay of carbon-14 [12]. Although the counting-efficiency for tritium is extremely low, the reproducibility of the method is excellent.

4 Amberlite IR120-H, Rohm & Haas Co., Philadelphia, Pa., and Duolite A–4, Chemical Process Co., Redwood City, Calif.

5 Supplied by Barnstead Still & Sterilizer Co., Boston, Mass.
